# Innovative technique to cover exposed dura mater after occipital osteomyelitis, using our triple layer using matriderm. A case report

**DOI:** 10.3389/fsurg.2026.1788499

**Published:** 2026-06-23

**Authors:** Ezzat E. E. Gabar, Mohammed Shawir, Mohammed Elnibras, Hytham Ibrahim Shokry Elatrozy, Mahmoud Shadad, Marei Al amari, Eyad Ali Faizo, Nadeem Muneer M. Maliabari

**Affiliations:** 1 Department of Surgery, King Khalid Hospital, Tabuk, Saudi Arabia; 2 Department of Surgery, College of Medicine, University of Tabuk, Tabuk, Saudi Arabia; 3 Consultant, Khartoum, Sudan

**Keywords:** flap, (STSG), galea, matriderm®, rotation, transposition, flap., transposition flap

## Abstract

Reconstructing a large, full-thickness cranial defect caused by trauma, with exposed dura mater, can be very challenging when using free or regional flaps. The wide current international practice is to employ either free flaps in fit patients or regional flaps in the form of Dermal Regeneration Templates (DRTs) in unfit patients, which can provide an alternative solution to flap surgery. In our study, we developed a new three-layer technique using a single-stage salvage procedure that combines Matriderm® dermal substitute (MedSkin Solutions Dr. Suwelack AG, Germany), galea rotation flap, and single pedicle fasciocutaneous transposition flap, which helps avoid complications associated with free flaps, such as microvascular-related thrombosis, pedicle twisting, and flap necrosis. We have covered the donor site with Matriderm® and a split-thickness skin graft (STSG). We here report the case of a 35-year-old male with post-traumatic occipital osteomyelitis, with scarring alopecia. In our new techniques, we employed a three-layer, single-stage salvage procedure, resulting in satisfactory functional and aesthetic outcomes and patient satisfaction while avoiding the time and complications of free flaps in medium-sized defects.

## Introduction

The intricate requirements of patients, frequently exceeding the capabilities of standard treatments, present a considerable reconstructive challenge for large scalp defects, rendering free flaps the most dependable option (1). Despite the 95% success rate of free flaps, 5% of these procedures fail due to various factors. There are three main types of risk factors for free flap failure: epidemiological variables, comorbidities, and other factors. The most important of these is venous thrombosis. Epidemiological risk factors encompass sex and age (2). Furthermore, peripheral vascular disease, coronary artery disease, diabetes, and hyperlipidaemia have been identified as comorbidities influencing free flap failure (3, 4). Neo-adjuvant therapies, including irradiation and chemotherapy, the use of anticoagulants, and operation duration are risk factors for free flap failure in head and neck reconstruction (5, 6). Due to problems associated with free flaps, we recommend our innovative one-stage, three-layer approach for patients deemed unfit or unsuitable for free transfer.

## Case report

**Figure 1:**
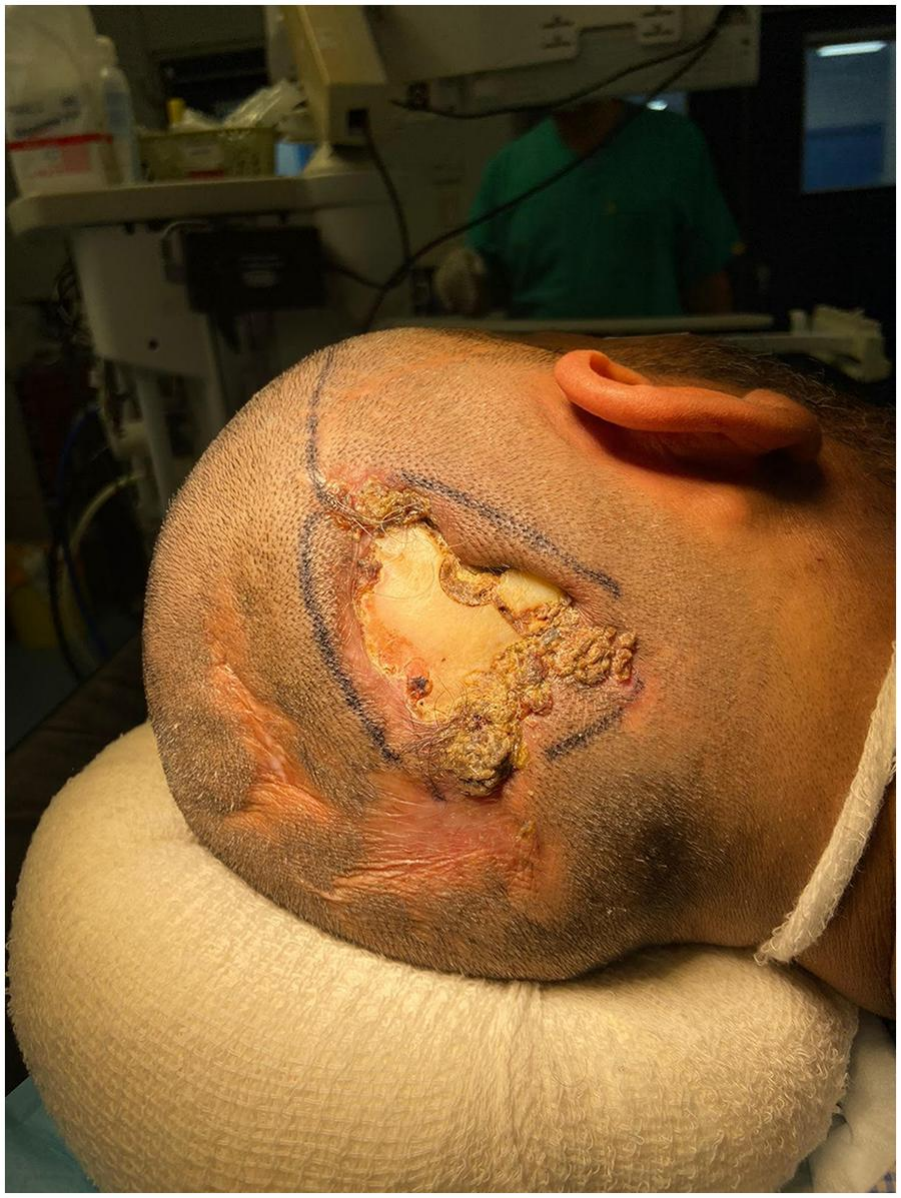
Occipital osteomyelitis after a motor vehicle collision.

A 35-year-old male presented to our plastic surgery outpatient clinic at King Khalid Hospital (KKH) with occipital osteomyelitis after a motor vehicle collision five years ago as a part of multi-trauma (Figure 1). His trauma five years ago consisted of a friction burn and laceration of the occipital area with significant skin loss. He has been managed by the attending neurosurgeon with a conservative approach. Nonetheless, he failed to attend his neurosurgical clinic during these five years, resulting in a neglected chronic osteomyelitic occipital ulcer with significant skin loss associated with scarring alopecia in the same area. After these five years, the patient decided to go back to his neurosurgeon clinic, who reviewed the case with an initial plan to excise the osteomyelitic bone and replace it with a bone graft. Further consultation between our plastic surgical team and the neurosurgical team led to an initial assessment of whether to cover the lost tissue or refer the patient to a tertiary centre. After thorough discussion among the neuroplastic team, we proceeded with preparing the patient for exploration and excision of the osteomyelitic bone. The preoperative work included kidney function tests, liver function tests, chest x-rays, ECG, and blood grouping and save, which were all normal. We proceeded with exploration after correction of anaemia with preoperative blood transfusion, and preoperative work with magnetic resonance imaging and computerised tomography was normal, albeit for osteomyelitic occipital bone. Our patient was informed about the procedure and signed a pre-operative consent.

**Figure 2:**
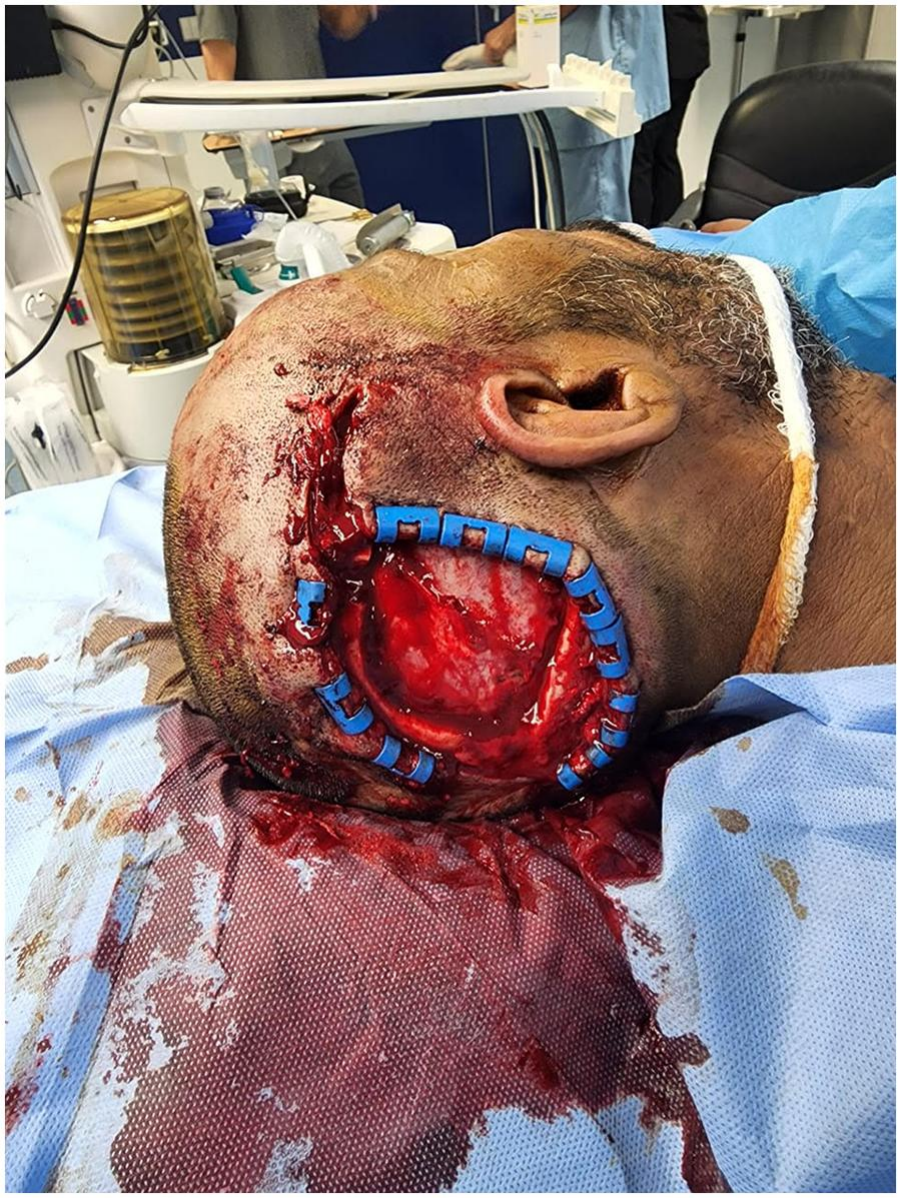
The neurosurgeon excised the osteomyelitic occipital bone.

The neurosurgeon excised the osteomyelitic occipital bone (Figure 2), micro-burring of the cortical bone was performed, and the dura was exposed without any coverage, with no way to cover it with bone graft at that time. We have used a layer of Matriderm® dermal substitute with its modified dimensions of 10 mm and 1 mm thick over the defect to give more protection to the brain tissue. We decided to rotate the entire galea rotation flap to cover the defect. On top of that we added Matriderm® dermal substitute, which was being soaked with 0.9% normal saline. The size of our Matriderm we used was 105 × 148 mm. Thereafter, a single pedicle transposition fasciocutaneous flap was raised and inset into the cranial defect measuring 55 centimetres in total surface area.

**Figure 3:**
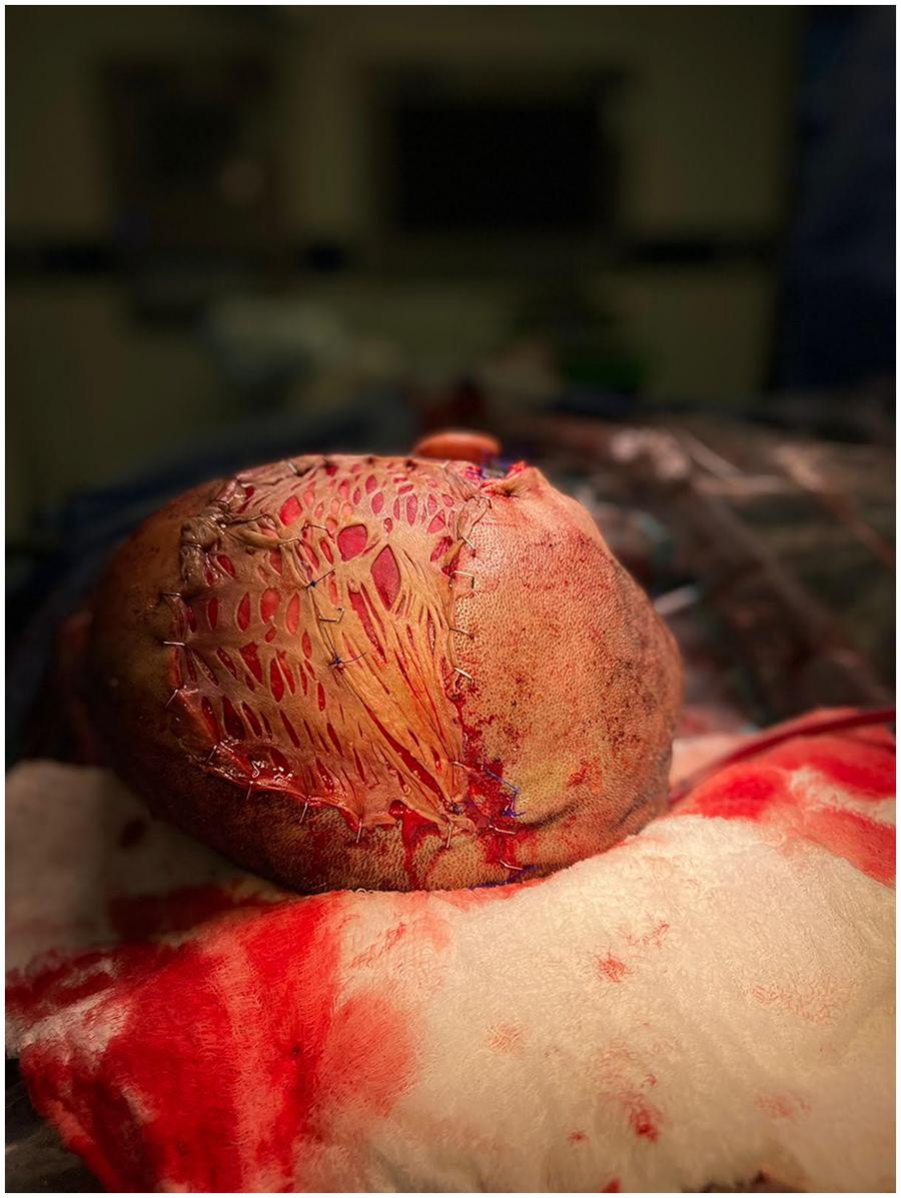
A triple layer of Matriderm, a galea rotation flap, and a single pedicle fasciocutaneous transposition flap (red arrow). The secondary defect was covered with Matriderm and STSG.

Dissection involved raising the hair-bearing frontoparietal transposition flap. Therefore, we ended up with three layers over the exposed dura composed of a galea rotation flap, Matriderm, and a single pedicle fasciocutaneous transposition flap (the flap was secured with 5/0 Vicryl all around the Matriderm). The frontal secondary defect was covered with Matriderm® dermal substitute. The Matriderm was covered with a split-thickness skin graft (STSG), which was harvested from the right thigh area. (STSG) was matched with a No. 11 scalpel (Figure 3). The donor site was covered with a closed multi-layer skin dressing. The STSG was covered with staples after securing haemostasis.

**Figure 4:**
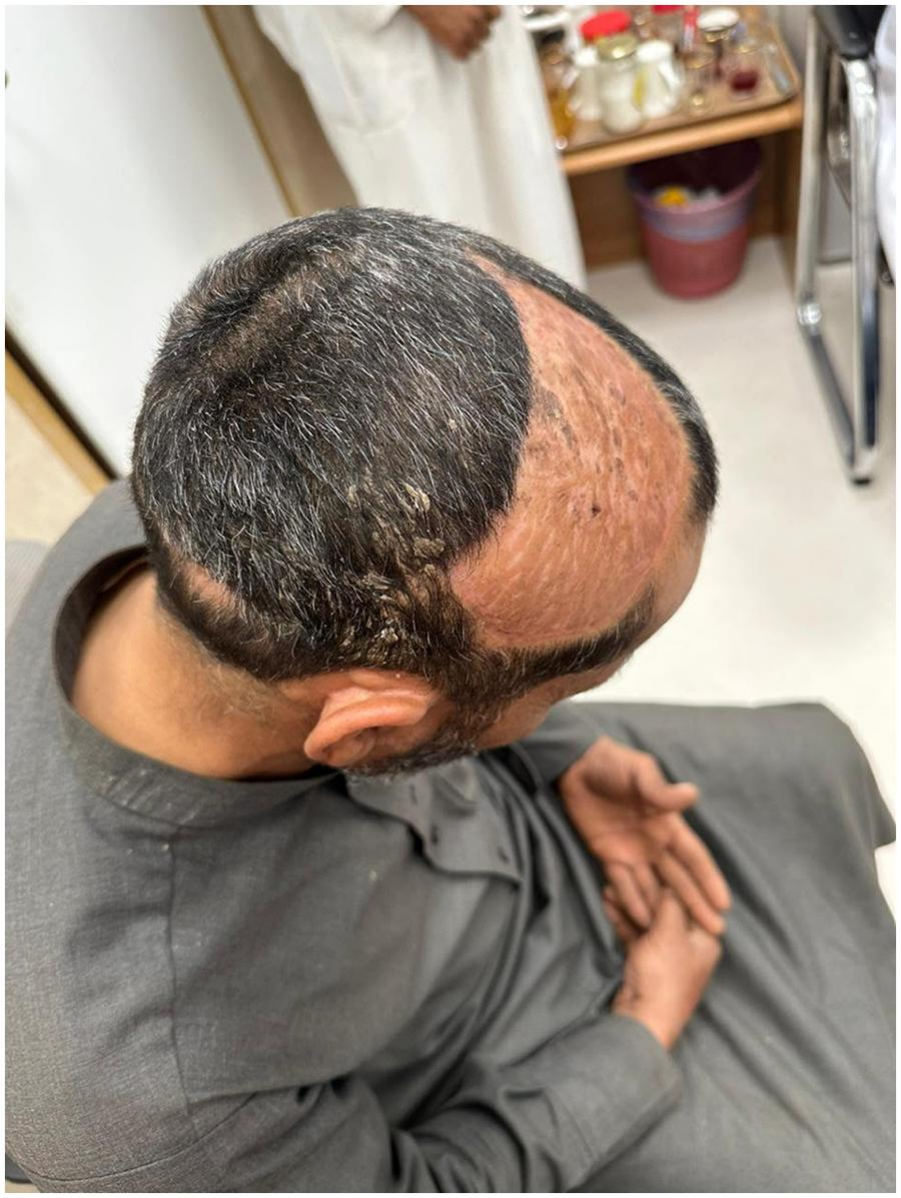
The patient was satisfied with the aesthetic outcome three months post-operatively.

**Figure 5:**
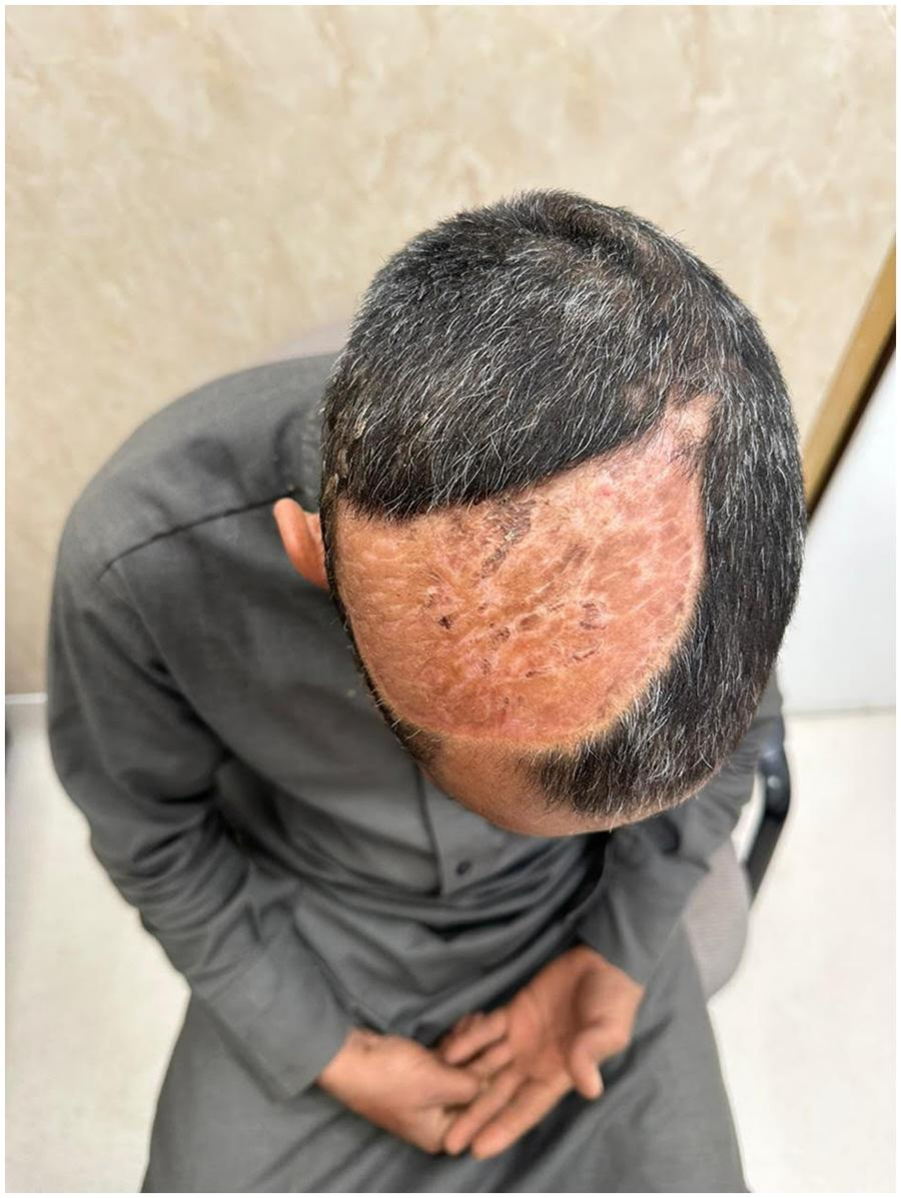
Three months postoperative with an aesthetic outcome.

The patients underwent a regular post-operative follow-up at 1, 2, and 3 weeks (as well as at 1, 3, and 6 months after the procedure) (Figure 4). The donor site from the right thigh had no morbidity. After six months, the neurosurgeon arrived at the conclusion that no further intervention was needed in terms of bone grafts. After six months, the patient was offered a tissue expansion to replace his frontal baldness area. However, the patient denied any more surgery, as he declared that he was fully satisfied. From the patient's perspective, three years after the operation, we had a 20-minute telephonic call with him. He denied any neurological symptoms pertaining to postural headaches. He denied experiencing any neurological deficits such as weakness or numbness. He did not report any symptoms of cognitive impairment, mental changes, lethargy or Seizures. The patient did not experience visual issues such as diplopia or vertigo (Figure 5).

## Discussion

Dermal-regenerating templates (DRTs) using Metriderm and STSGs can provide an alternative solution to flap surgery, especially in cases of failed free flaps (7). Using two stages, separated by an interval of 3–6 weeks, employing acellular dermal matrices (Integra) and vacuum-assisted closure (VAC) has been described as a suitable alternative in complex cranial reconstructions after oncological resection and post-traumatic defects with exposed bone or dura (8, 9). However, the use of the single-stage, two-layer technique combining Matriderm® dermal substitute and STSG is superior to the two-stage procedure as described by Vilela et al. (10). Giovanni et al., in his 16-patient retrospective study, has concluded that the use of Matriderm® and split-thickness skin grafting for scalp full-thickness defects reconstruction resulted in an optimal, stable, and safe procedure, suitable for elderly patients (11). We did not incorporate Giovanni and his Vancouver Scar Scale score (VSS) due to the nature of our study being a case report, whereas his is a retrospective study (11). The Vancouver Scar Scale (VSS) has four parameters: vascularity (0–3), pigmentation (0–2), pliability (0–5), and height (0–3). Recall that a lower score indicates superior scar condition and quality (11). Despite the novel and promising nature of our unique one-stage, three-layer technique, a comprehensive securitisation process, such as a future retrospective or cohort study, is essential to validate our assertion, highlighting the limitations of our research. We used Matriderm®, a very porous membrane made from freeze-dried material, as a dermal matrix with a thickness of 1 or 2 mm; it consists of dried acellular collagen, which is composed of hydroxylated elastin and bovine cutaneous collagen types I, III, and V (12). Dermal substitutes function as collagen scaffolds for fibroblasts, facilitating neovascularisation and accelerating cell migration (13). The histological phases of dermal regeneration following the implantation of a dermal substitute encompass four stages: scaffold imbibition, fibroblast migration, neovascularisation, and remodelling, culminating in final maturation as Matriderm® is resorbed within six weeks, subsequent to collagen production induced by fibroblasts (14, 15).

We endorse our novel single-stage, three-layer approaches (Galea rotation flap, Matriderm, and single pedicle transposition flap), as they offer solid coverage for extensive cranial lesions, circumventing the complications and delays associated with free flap methods. Nevertheless, Sooyeon Park has achieved comparable favourable outcomes by utilising his bilayer approaches (Matriderm, STSG, and full-thickness skin graft FTG) without necessitating a craniotomy in older patients with angiosarcoma (16). Scalp defects are generally classified as small (up to about 10 cm^2^), medium (10–20 cm^2^), and large (over 20 cm^2^) (17–19). Deepak Krishna et al. conducted a retrospective analysis of 54 cases, employing various procedures to address full scalp defects of diverse aetiologies. These procedures included local flaps, rotation flaps with primary closure, transposition flaps with STSG, single rotation flaps, one double rotation flap, and one free latissimus dorsi muscle flap. Although the aesthetic outcomes in all cases were commendable, none of the techniques presented were comparable to our innovative single-stage, three-layer approach for managing full-thickness medium-sized defects (20). Furthermore, our single-stage, three-layer technique has yielded a stable, durable, functional, and aesthetically pleasing outcome, as inadequate coverage of a full-thickness defect may precipitate osteomyelitis of the pericranium and severe neurological complications, including meningitis and brain abscess, due to the potential for infection from the scalp wound to access the cranial cavity via valveless emissary veins (21). During the preoperative phase, it is imperative to choose coverage approaches based on critical parameters, such as size, location, defect characteristics, radiation requirements, the presence of hair on the skin, the condition of surrounding tissue, and the likelihood of hairline distortion (22–24). The donor site for transposition flaps should be in a less aesthetically prominent region, enabling adjacent long hairs to conceal the skin (25). In this instance, the patient expressed satisfaction with the balding region of his frontal scalp, despite our proposal of an expander operation to address his bald area, which he finally declined. We utilised a dermal regenerating template (Matriderm) following the machining of the outer table to enhance the aesthetic result and longevity of STSG (26). According to van Driel et al., small to medium-sized full cranial bone defects (≤5–7 cm) can be left without replacement if not located at the forehead or occipital region, which are cosmetic and pressure-sensitive areas, respectively (27). They also recommended morselised bone grafts for small-size defects and or rib grafts for medium-size defects. Large-sized bone defects require a vascularised rib graft with a free latissimus dorsi muscle flap cover (28). As of the writing of this article, our novel technique for addressing a full-thickness calvaria defect measuring 55 cm in total surface area, employing a triple layer of a galea rotation flap, Matriderm, and a transposition fasciocutaneous flap at the recipient site, in conjunction with Matriderm and STGS at the donor site—remains undocumented in the literature. Our patient did not experience bone resorption or infection, the predominant complications after cranioplasty that necessitate revision surgery (29). Comparison with Alternative Dermal Substitutes (e.g., Integra):

The application protocol for Matriderm involves a single-stage operation (instant STSG), thereby minimising operating periods, while Integra often necessitates a two-stage method due to its silicone covering that must be excised prior to grafting.

Matriderm is a monolayer matrix consisting of bovine collagen types I, III, and V, in addition to elastin hydrolysate, which offers a natural scaffold that facilitates swift cellular penetration. Integra comprises bovine tendon collagen and glycosaminoglycan, i.e., composition and structure. Matriderm is quickly absorbed (usually within weeks), but Integra, which is cross-linked, may take months to years to fully integrate and be replaced by the patient's own tissue. This is known as the resorption rate. Matriderm, owing to its elastin composition, is frequently linked to enhanced pliability and superior aesthetic results in dynamic regions like the scalp, i.e., clinical performance. Research demonstrates that Matriderm presents a reduced chance of total lysis (failure) relative to some alternatives, such as Integra, in full-thickness wounds (i.e., scar quality and flexibility). Matriderm capacity to be placed concurrently with a split-thickness skin graft (STSG) facilitates a streamlined, one-step repair for extensive scalp tumour excisions, i.e., efficiency. Matriderm facilitates optimal aesthetic and functional outcomes by ensuring adequate vascularity and pigmentation, resulting in results that closely resemble healthy skin. It is regarded as manageable, and it adheres effectively to the vascularised wound bed (30).

We concur with our neurosurgeon's assessment that the patient faced a danger of latent infection (osteomyelitis), hence a non-absorbable cranioplasty prosthesis was not employed in this procedure. The patient's primary concern was the unsightly skin defect revealing damaged bones. After our triple-layer technique resolved the issue, the patient declined additional surgery for cranioplasty. Due to his refusal of cranioplasty, we did not solicit a postoperative brain CT. Utmost care was exercised, and no durotomy was observed during our procedure. Numerous publications have documented the application of Matriderm on both the exposed brain and the intact dura, reporting no problems and outstanding cosmetic outcomes (10, 31).

## Conclusion

The combination of Galea rotation, Matriderm, and a single pedicle transposition fasciocutaneous flap effectively addresses medium-sized 55 square centimetre calvaria defects. Calvaria defects with exposed dura mater, when addressed using our single-stage, three-layer technique, provide enhanced durable coverage and efficacy compared to free flap transfer. This method is more efficient than free flap transfer, which is often time- and labour-intensive and carries risks, such as microvascular thrombosis. Additionally, there are complications at the recipient site, including flap necrosis and flap failure.

### Limitation and recommendation

Despite our initially promising and encouraging short-term results, the long-term results require further investigation through future cohort studies to scrutinise them. The ability of our coverage to endure high doses of post-reconstructive radiation in cases of scalp cancers remains uncertain. The durability and longevity of this new technique necessitate extensive long-term studies, particularly in the forehead and occipital regions, which are both cosmetic and pressure-sensitive areas. To address these long-term enquiries, it is essential to persist with our novel technique and critique our results.

## Data Availability

The original contributions presented in the study are included in the article/supplementary material, further inquiries can be directed to the corresponding authors.
